# Stereo‐electroencephalography–guided responsive neuromodulation: A propensity‐matched cohort study

**DOI:** 10.1002/epi.70377

**Published:** 2026-07-14

**Authors:** David Botros, Julian Brown, John D. Rolston, Paul A. House, Amir Arain, Matthew Jensen, Blake Newman, Brian Johnson, Sindhu Richards, Angela Peters, Shervin Rahimpour, Ben Shofty

**Affiliations:** ^1^ Department of Neurosurgery, Clinical Neurosciences Center University of Utah Salt Lake City Utah USA; ^2^ Spencer Fox Eccles School of Medicine, University of Utah Salt Lake City Utah USA; ^3^ Department of Neurosurgery Mass General Brigham, Harvard Medical School Boston Massachusetts USA; ^4^ Neurosurgical Associates, LLC Salt Lake City Utah USA; ^5^ Department of Neurology University of Utah Salt Lake City Utah USA

**Keywords:** closed‐loop stimulation, drug‐resistant epilepsy, patient‐reported outcomes, seizure severity

## Abstract

**Objective:**

The optimal pre‐implantation evaluation and surgical targeting strategies for responsive neurostimulation (RNS) in patients with unresectable, drug‐resistant focal epilepsy continue to be debated. Although stereo‐electroencephalography (sEEG) is central to characterizing the seizure‐onset zone, its role in preoperative testing for neuromodulation remains undefined. We examined whether sEEG‐guided targeting for RNS implantation confers seizure‐reduction benefit compared with RNS implantation without sEEG.

**Methods:**

We retrospectively reviewed patients who underwent RNS implantation at our institution in 2015–2024. Patients were propensity score–matched based on demographics and epilepsy characteristics to create balanced groups of patients with and without prior sEEG. The primary outcome measure was seizure reduction as a percent change from baseline frequency, determined using linear regression.

**Results:**

Of 99 patients (median ± standard error [SE] follow‐up 45.3 ± 2.9 months), 78 (79%) underwent sEEG monitoring before RNS implantation and 21 (21%) did not. Most patients (62%) had no prior interventions for epilepsy and had non‐lesional epilepsy (75%). Mean seizure reduction at last follow‐up was 72.4% ± 29.3%. There were 21 patients in each propensity‐matched group. The use of preoperative sEEG was not associated with greater seizure reduction in the original (*p* = .835) and propensity score–matched cohorts (*p* = .914). In the unmatched cohort, female sex (*β* = 16.7, *p* = .006,) older age (*β* = .55, *p* = .027), thalamic RNS stimulation (*β* = 18.0, *p* = .030), and follow‐up duration (*β* = .2, *p* = .029) were associated with greater seizure reduction on linear regression. Subgroup analyses among epilepsy subtypes (bitemporal, temporal, or extratemporal) found no statistically significant differences in seizure reduction.

**Significance:**

In a climate of growing delays in epilepsy care, we did not observe differences in RNS efficacy with or without prior sEEG monitoring in patients with unilateral or bilateral temporal lobe epilepsy, temporal‐plus epilepsy, and extratemporal epilepsy. Although prospective data are needed to investigate the relative benefit of preoperative sEEG use, these findings may help guide patient selection and optimize definitive surgical planning.

**Key points**

- Preoperative stereo‐electroencephalography (sEEG) was not associated with greater long‐term seizure reduction after responsive neurostimulation (RNS).
- Long‐term seizure outcomes were comparable with RNS targeting guided by sEEG or scalp video‐EEG alone.
- Early seizure reduction favored sEEG, but differences resolved with longer follow‐up.

## 1 INTRODUCTION

Drug‐resistant epilepsy (DRE) affects a substantial proportion of patients with epilepsy and is associated with significant morbidity, mortality, psychosocial burden, and lower quality of life.^1^, ^2^ Neuromodulation strategies have become a critical component of the treatment landscape for patients who are not candidates for resection or ablation. Responsive neurostimulation (RNS) is an invasive treatment that delivers closed‐loop electrical stimulation in response to detected epileptiform activity via a depth electrode or subdural strip array connected to a cranially mounted internal pulse generator. RNS has demonstrated long‐term efficacy in reducing seizure frequency and improving quality of life for appropriately selected patients.^3^

Despite the growing adoption of RNS, optimal strategies for pre‐implantation evaluation in different epilepsy subtypes remain uncertain. Stereo‐electroencephalography (sEEG) is an important tool in the preoperative evaluation of patients with DRE, often facilitating seizure‐focus resection, disconnection, or ablation.^4^ Although sEEG provides high‐resolution data, it requires an additional invasive procedure—with associated risks, cost, and patient burden—and it is unclear whether sEEG produces superior outcomes for neuromodulation techniques, specifically RNS.^5^

Previous studies of RNS have reported favorable outcomes overall, but few have specifically compared patients who underwent sEEG with those whose implantation was guided non‐invasively.^6^ Owens et al.^7^ reported that patients who underwent sEEG saw a clinical reduction in seizure burden more frequently than those who did not undergo sEEG, although this study was limited by sample size. Geller et al.^8^ posited that RNS benefit is likely related to sEEG‐informed precise electrode targeting. Conversely, Razavi et al.^9^ previously presented a cohort of 150 patients with similar seizure outcomes, irrespective of invasive intracranial monitoring. Furthermore, little is known about whether patient subgroups, such as those with structural lesions, prior epilepsy surgeries, distinct seizure subtypes, or differing medication burdens, derive disproportionate benefits from sEEG. Identifying these subgroups may help refine patient selection and improve the efficiency and safety of RNS planning.

In this retrospective review of patients who underwent RNS implantation at our institution, we compared long‐term outcomes between those who underwent preoperative sEEG and those whose implantation was not guided by invasive monitoring, with a focus on clinician‐reported seizure reduction. We used propensity score matching to create balanced groups based on the likelihood that patients would undergo sEEG investigation before definitive surgery and identified clinical features associated with improved outcomes to clarify the role of sEEG in optimizing surgical planning for RNS.

## 2 METHODS

### 2.1 Patient selection and data collection

We performed a retrospective review of all patients with drug‐resistant focal epilepsy, who underwent RNS implantation at our institution (a comprehensive Level 4 epilepsy center) or had RNS implantation elsewhere and were monitored clinically at our institution between September 2015 and October 2024. The included patients had at least 6 months of follow‐up data after implantation. Electronic medical records were reviewed for patient age, sex, duration of epilepsy, number of anti‐seizure medications (ASMs) used, seizure frequency per month, seizure semiology, prior interventions for epilepsy, time from initial neurosurgical consultation to RNS placement, sEEG implantation, and RNS stimulation type. Postoperative data collected included epileptologist‐documented seizure frequency and number of ASMs used at each follow‐up interval (1 and 12 months) and at last follow‐up. Patients were started on RNS at the 1‐month follow‐up interval. The Neuropace (Mountain View, California, USA) patient data management system was used to collect RNS parameters (such as current, pulse width, charge density, duration, and frequency).

Seizure subtype was identified via video‐electroencephalography (vEEG) during the initial hospitalization for epilepsy monitoring as unilateral temporal lobe epilepsy (TLE), bilateral TLE, unilateral neocortical temporal epilepsy, temporal‐plus epilepsy, or extratemporal epilepsy based on the interpretation of the attending epileptologist. Seizure frequency was captured from the epileptologist's electronic medical record documentation at each follow‐up visit, and seizure reduction was calculated at each follow‐up visit when compared with baseline seizure frequency. sEEG constructs and epileptologist interpretation were examined to identify patients with thalamic sampling and a broad neocortical seizure‐onset zones, respectively.

### 2.2 Propensity score matched analysis

Propensity score matching was performed using the “matchit” package in R to calculate the propensity of undergoing sEEG monitoring before RNS implantation. Propensity to undergo sEEG was calculated using age, sex, epilepsy duration, prior interventions, epilepsy subtype, number of preoperative ASMs, and follow‐up duration. Optimal, 1:1 matching was used to compare patients with and without sEEG and minimize differences between groups. Group balance was assessed using a Love plot to visualize standardized differences between groups (Figure S1).

### 2.3 Statistical analysis

Data were collected using Microsoft Excel (version 16.102.2). Statistical analyses were conducted in R version 4.4.2 (R Foundation for Statistical Computing, Vienna, Austria). Chi‐square and Fisher's exact testing were used for categorical variables where appropriate, and Mann–Whitney *U* testing was used for continuous non‐parametric data. Seizure reduction percentage at the last follow‐up was analyzed as a continuous outcome. Candidate predictors included age, sex, epilepsy history (epilepsy duration, epilepsy subtype, prior interventions, prior vagus nerve stimulation [VNS]), surgical characteristics (preoperative sEEG, thalamic lead placement, number of ASMs at implantation), and follow‐up duration. These statistical analyses were performed separately for the entire cohort and for the propensity‐score matched cohorts and presented in separate tables.

To prevent overfitting and account for potential multicollinearity, we used least absolute shrinkage and selection operator (LASSO) regression for variable selection using the *glmnet* package in R, with all predictors entered simultaneously for the entire cohort. Tenfold cross‐validation identified the optimal penalty parameter (λ_min). Predictors with non‐zero coefficients at λ_min were retained. The retained variables were entered into an unpenalized multivariable linear regression model to obtain *β* estimates, 95% confidence intervals (CIs), and *p*‐values. Model assumptions (linearity, residual distribution, and heteroscedasticity) were checked and met. Statistical significance was defined as *p* < .05.

## 3 RESULTS

Our cohort included 99 patients who were treated with RNS. Patients who underwent preoperative sEEG (*n* = 78) had a median age of 34.3 years (interquartile range [IQR] 17.1), whereas those without sEEG (*n* = 21) had a median age of 39.0 years (IQR 24.2) (Table 1). No difference in the proportion of male patients was observed (*p* = .174) between the sEEG group (56.4%) and the no‐sEEG group (38.5%). The median duration of epilepsy was 16.8 years (IQR 15) in the sEEG group and 20.5 years (IQR 21) in the no‐sEEG group (*p* = .284).

**TABLE 1:** Demographics and characteristics of patients with and without sEEG.

Variable	No sEEG (*n* = 21)	Preop sEEG (*n* = 78)	*p*
Age in years, mean (±SD)	39.0 (±15.0)	34.3 (±11.8)	.191
Male sex	10 (38.5)	44 (56.4)	.174
Epilepsy duration in years, median (±SE)	20.5 (±3.2)	16.8 (±1.3)	.284
Epilepsy characteristics			.324
FIC	3 (14.3)	8 (10.3)	
FPC	‐	2 (2.6)	
FBTC	17 (81.0)	68 (87.2)	
GTC	1 (4.8)	‐	
Prior interventions			.870
None	14 (66.7)	47 (60.3)	
1	6 (28.6)	23 (29.5)	
2	1 (4.76)	8 (10.3)	
Epilepsy subtype			.025*
Unilateral TLE	3 (14.3)	29 (37.2)	
Bilateral TLE	8 (38.1)	16 (20.5)	
Unilateral neocortical temporal	2 (9.5)	‐	
Temporal plus	4 (19)	18 (23.1)	
Extratemporal	4 (19)	15 (19.2)	
Lesion	9 (42.9)	16 (20.5)	.070
RNS type			.808
Unilateral	5 (23.8)	24 (30.8)	
Bilateral	11 (52.4)	39 (50.0)	
Thalamic	5 (23.8)	15 (19.2)	
Months from initial consult to RNS placement, median (±SE)	2.8 (±.4)	6.9 (±.6)	< .001*
Preoperative ASMs, mean (±SD)	2.5 (±.6)	2.5 (±.9)	.991
Postoperative ASMs, mean (±SD)	2.5 (±.8)	2.5 (±.9)	.776
Follow‐up duration in months, median (±SE)	45.0 (±6.6)	51.3 (±3.2)	.381
Monthly seizure reduction at last follow‐up, mean % (±SEM)	74 (±6.1)	72 (±3.4)	.777
RNS parameters, mean (±SD)^a^			
Current (mA)	4.84 (2.32)	4.49 (2.05)	.533
Pulse width (μs)	160 (.0)	159 (4.56)	.320
Charge density (μC/Cm^2^)	2.80 (1.1)	2.75 (.81)	.856
Duration (ms)	762 (1045)	536 (1091)	.391
Frequency (Hz)	172 (50.7)	174 (57.4)	.901

*Note*: Values are *n* (%) unless otherwise indicated.

Abbreviations: ASM, anti‐seizure medication; Cm, centimeters; FBTC, focal‐to‐bilateral tonic–clonic seizure; FIC, focal impaired consciousness seizure; FPC, focal preserved consciousness seizure; GTC, generalized tonic–clonic; Hz, hertz; μs, microsecond; mA, milliampere; ms, millisecond; preop, preoperative; RNS, responsive neurostimulation; SD, standard deviation; SE, standard error; sEEG, stereo‐electroencephalography; SEM, standard error of the mean; TLE, temporal lobe epilepsy.

* Statistically significant (*p* < .05).

^a^ Data were collected via the Neuropace patient data management system.

Most patients (73.1% in the sEEG group and 76.2% in the no‐sEEG group) had focal to bilateral tonic–clonic seizures. Other seizure types were less common, with small numbers of patients experiencing focal impaired consciousness seizures alone, focal preserved consciousness seizures, or generalized tonic–clonic seizures.

Most patients had no prior surgical interventions for epilepsy (60.3% sEEG vs 66.7% no‐sEEG, *p* = .870); smaller proportions of patients had one (29.5% vs 28.6%) or two (10.3% vs 4.8%) prior interventions. Specifically, prior VNS was also similar across groups (*p* = .650). The distribution of epilepsy subtypes (typified using scalp EEG as recorded during long‐term monitoring admission) differed (*p* = .025), with unilateral TLE being more common in the sEEG group (37.2% vs 14.3%) and bilateral TLE more frequent in the no‐sEEG group (20.5% vs 38.1%). Among patients with unilateral TLE, epilepsy arising from the dominant temporal lobe did not differ between the groups (40% sEEG vs 67% no‐sEEG; *p* = .559). The number of temporal plus, extratemporal, and lesional cases were similar in both groups. Patients with unilateral TLE underwent neuromodulation most often (46%) because of involvement of the dominant temporal lobe followed by high concern for bilateral TLE (38%).

The no‐sEEG patients were most frequently recommended for upfront RNS because of reliable bilateral focal onset of seizure activity (8 [38%] bitemporal; 4 [19%] bifrontal), followed by declining intracranial monitoring (4 [19%]). Patients who declined intracranial monitoring often cited a lack of desire for an additional hospitalization or had dominant TLE and were not interested in resective therapy and the preceding investigative sEEG. Three patients (14%) failed prior surgical interventions, and RNS was recommended after multidisciplinary conference. Two (9.5%) patients with Phase 1 concomitant unilateral dominant TLE declined laser ablation. Notably, median time from initial neurosurgical consultation to RNS placement was significantly longer in patients who underwent sEEG monitoring (6.9 months for sEEG patients vs 2.8 months for no‐sEEG patients, *p* < .001).

RNS depth electrode or electrode strip location varied widely among patients and details of patient‐specific constructs are included in Table S1. Unilateral RNS stimulation was performed in 5 patients (23.8%) in the no‐sEEG group and 24 (30.8%) in the sEEG group, bilateral RNS stimulation in 11 (52.4%) and 39 (50%), respectively, and thalamic RNS stimulation in 5 (23.8%) and 15 (19.2%), respectively. Stimulation location did not differ significantly among groups (*p* = .808). Among patients in the sEEG group, at least one seizure‐onset zone was successfully identified in 74 patients (95%); only 8 (10%) had thalamic sampling as part of their sEEG construct, and 16 (21%) patients were found to have a broad neocortical seizure‐onset zone. Patients who were found to have a broad neocortical seizure‐onset zone were more likely to undergo thalamic RNS implantation (odds ratio [OR] 1.86, *p* = .0038), whereas there was no association between thalamic sampling as part of the sEEG construct and final thalamic RNS implantation (*p* = .225). Postoperatively, our cohort experienced 4 complications (4%). One patient each experienced a surgical site infection requiring explantation, a lead fracture requiring replacement, significant headaches requiring lead replacement, and a postoperative pulmonary embolism.

The mean numbers of preoperative and postoperative ASMs taken were similar between the sEEG and no‐sEEG patients (2.5 ± .9 vs 2.5 ± .6, *p* = .991, preoperatively; 2.5 ± .9 vs 2.5 ± .8, *p* = .776, postoperatively). The median follow‐up duration was 51.3 months (standard error [SE] 3.2) for sEEG patients and 45.0 months (SE 6.6) for no‐sEEG patients (*p* = .381). The mean monthly seizure reduction (at last follow‐up) was 72% (standard error of the mean [SEM] 3.4) in the sEEG group and 74% (SEM 6.1) in the no‐sEEG group (*p* = .777). Seizure reduction also appeared to increase over time without any group differences (Figure 1A). Stimulation parameters did not differ between sEEG and no‐sEEG patients (Table 1, *p* > .05) or by epilepsy subtype (*p* > .05).

**FIGURE 1:**
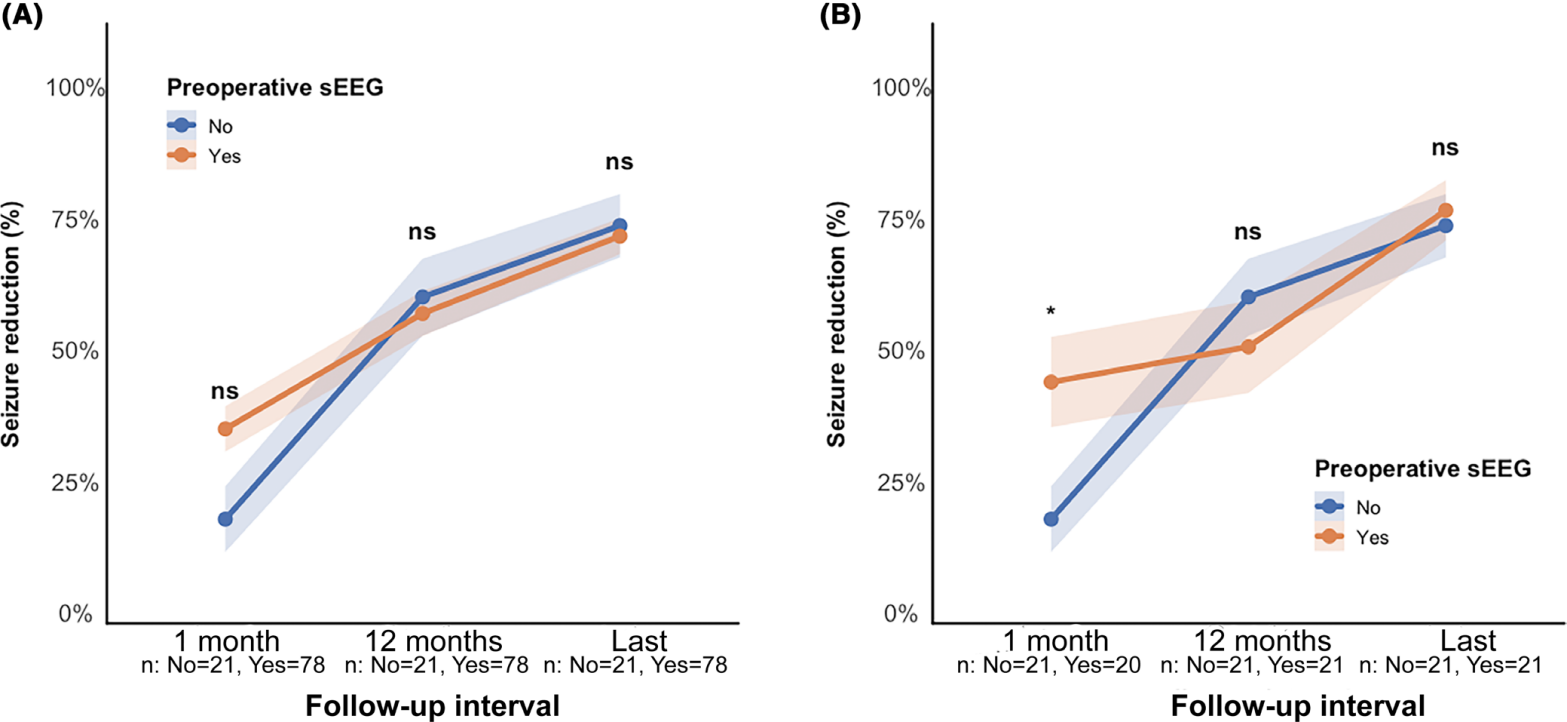
Seizure reduction percentage at each follow‐up interval for the entire cohort (A) and the propensity score–matched cohort (B) with error bars denoting the standard error of the mean. (*p < .05; ns, Not significant).

### 3.1 Univariate and multivariable regression

Simple linear regression across our entire cohort demonstrated an association of female sex (*β* = 17.0, *p* = .003) with greater seizure reduction (Table 2). Age (*p* = .072), epilepsy duration (*p* = .844), prior interventions (one intervention, *p* = .849; two interventions *p* = .365), prior VNS placement (*p* = .464), preoperative sEEG use (*p* = .835), the presence of a lesion (*p* = .899), time from initial consultation to RNS placement (*p* = .756), preoperative ASM (*p* = .276), unilateral RNS (*p* = .563), thalamic RNS (*p* = .299), and follow‐up duration (*p* = .102) were not associated with seizure reduction on univariate regression.

**TABLE 2:** Linear regression for % seizure reduction.

Variable	Univariate *β* (±95% CI)	*p*	Multivariate *β* (±95% CI)	*p*
Age, years, increasing	.4 (−.04 to .9)	.072	.5 (.1 to 1.0)	.027*
Female, ref: male	17 (5.9 to 28)	.003*	16.7 (5.1 to 28.3)	.006*
Epilepsy duration, years, increasing	−.1 (−.5 to .4)	.844	−.4 (−.9 to .1)	.115
Prior interventions, ref: none				
1	1.2 (−12 to 14)	.849	‐	‐
2	−9.6 (−30 to 11)	.365	‐	‐
VNS	4.9 (−8.3 to 18)	.464	13.6 (−.8 to 27.9)	.068
Preoperative sEEG	−1.5 (−16 to 13)	.835	‐	‐
Lesion	−.9 (−14 to 13)	.899	‐	‐
Epilepsy subtype, ref: unilateral TLE				
Bilateral TLE	13 (−2.8 to 28)	.108	8.7 (−6.8 to 24.1)	.274
Unilateral neocortical TLE	32 (−9.9 to 74)	.132	25.8 (−12.5 to 64.0)	.189
Temporal plus	15 (−.5 to 31)	.058	7.7 (−8.3 to 23.7)	.349
Extratemporal	2.8 (−13 to 19)	.733	−2.1 (−18.7 to 14.3)	.800
Time from initial consult to RNS placement, months, increasing	.13 (−.7 to 1.0)	.756	‐	‐
Preoperative ASM, increasing	−3.9 (−11 to 3.2)	.276	−6.6 (−13.9 to .8)	.083
RNS type, ref: bilateral				
Unilateral	−4.0 (−11.0 to 3.0)	.563	−5.1 (−19.6 to 9.4)	.493
Thalamic	8.0 (−7.0 to 23.0)	.299	18.0 (2.0 to 34.0)	.030*
Follow‐up duration, months, increasing	.2 (−.03 to .37)	.102	.2 (.03 to .4)	.029*

*Note*: Akaike information criterion (multivariable model): 33.02.

Abbreviations: ASM, anti‐seizure medication; CI, confidence interval; RNS, responsive neurostimulation; sEEG, stereo‐electroencephalography; TLE, temporal lobe epilepsy; VNS, vagus nerve stimulator.

* Statistically significant (*p* < .05).

In the multivariable LASSO model, greater seizure reduction was significantly associated with older age (*β* = .5, 95% CI: .1–1.0, *p* = .027), female sex (vs male; *β* = 16.7, 95% CI: 5.1–28.3, *p* = .006), thalamic RNS stimulation (*β* = 18.0, 95% CI: 2.0–34.0, *p* = .030), and longer follow‐up duration (*β* = .2, 95% CI: .03–.40, *p* = .029).

Variables that were included in the final model but were not statistically significant included epilepsy duration, epilepsy subtype, prior VNS implantation, and number of ASMs at the time of surgery.

### 3.2 Propensity score matched analysis

Given the ongoing clinical equipoise regarding the use of sEEG for varying epilepsy subtypes and the inability to conduct randomized controlled trials centered on sEEG use, we elected to create propensity score–matched cohorts for further analysis. Propensity score matching produced well‐balanced groups of 21 patients in the sEEG and no‐sEEG groups (Table 3). Of note, there was no evidence of a difference in the number of patients with the scalp EEG‐determined epilepsy subtype, the number of prior interventions, and epilepsy duration between groups. At 1‐month follow‐up, seizure reduction among the no‐sEEG group was lower than in those who underwent preoperative sEEG (18% vs 44%, *p* = .027) (Figure 1B). At last follow‐up, however, seizure outcomes were similar (*p* = .914), with sEEG patients reporting 75% (SEM 6.1) seizure reduction and no‐sEEG patients reporting 74% (SEM 6.1) seizure reduction. Among the propensity score–matched cohorts, RNS stimulation parameters were also similar between sEEG and no‐sEEG patients (Table 3, *p* > .05) as well as by epilepsy subtype (*p* > .05). Time to RNS from initial consult was longer in the sEEG group (6.3 months for the sEEG group vs 2.8 months for the no‐sEEG group, *p* < .001). The number of ASMs at last follow‐up (*p* = .360) and patient‐reported seizure severity (*p* > .999) were also similar between groups. Multivariable LASSO regression using variables not included in propensity score generation did not reveal any significant associations with seizure reduction. Of note, patient sex, age, and follow‐up duration were all used for propensity score matching and, therefore, were not included in multivariable analyses. Thalamic RNS, which was significantly associated with increased seizure reduction in the entire cohort, was not associated with seizure reduction in the propensity score–matched cohort.

**TABLE 3:** Propensity‐score matched groups with and without preoperative sEEG.

Variable	No sEEG (*n* = 21)	Preop sEEG (*n* = 21)	*p*
Age in years, mean (±SD)	39.0 (±15.0)	37.2 (±13.2)	.684
Male sex	10 (47.6)	10 (47.6)	> .999
Epilepsy duration in years, median (±SE)	20.5 (3.2)	20.7 (2.8)	.965
Epilepsy characteristics			.343
FIC	3 (14.3)	1 (4.8)	
FPC	‐	‐	
FBTC	17 (81.0)	20 (95.2)	
GTC	1 (4.8)	‐	
Prior interventions			> .999
None	14 (66.7)	15 (71.4)	
1	6 (28.6)	6 (28.6)	
2	1 (4.76)	‐	
Epilepsy subtype			.793
Unilateral TLE	3 (14.3)	4 (19.0)	
Bilateral TLE	8 (38.1)	7 (33.3)	
Unilateral neocortical temporal	2 (9.5)	‐	
Temporal plus	4 (19.0)	5 (23.8)	
Extratemporal	4 (19.0)	5 (23.8)	
Lesion	9 (42.9)	9 (42.9)	> .999
RNS type			.775
Unilateral	5 (23.8)	5 (23.8)	
Bilateral	11 (52.4)	13 (61.9)	
Thalamic	5 (23.8)	3 (14.3)	
Months from initial consult to RNS placement, median (±SE)	2.8 (1.7)	6.3 (3.3)	< .001*
Preoperative ASMs, median (±SE)	2.5 (.6)	2.3 (.9)	.448
Postoperative ASMs, median (±SE)	2.5 (.8)	2.3 (.8)	.360
Follow‐up duration in months, median (±SE)	45.0 (6.7)	56.7 (7.2)	.244
Monthly seizure reduction at last follow‐up, mean % (±SEM)	74 (6.1)	75 (6.1)	.914
RNS parameters, mean (±SD)^a^			
Current, mA	4.84 (2.32)	4.82 (2.23)	.978
Pulse width, μs	160 (.0)	158 (8.73)	.329
Charge density, μC/cm^2^	2.80 (1.1)	2.97 (.73)	.555
Duration, ms	762 (1045)	571 (1262)	.597
Frequency, Hz	172 (50.7)	181 (35.3)	.523

*Note*: Values are *n* (%) unless otherwise indicated.

Abbreviations: ASM, anti‐seizure medication; FBTC, focal‐to‐bilateral tonic clonic seizure; FIC, focal impaired consciousness seizure; FPC, focal preserved consciousness seizure; GTC, generalized tonic–clonic; preop, preoperative; RNS, responsive neurostimulation; SD, standard deviation; SE, standard error; sEEG, stereo‐electroencephalography; SEM, standard error of the mean; TLE, temporal lobe epilepsy.

* Statistically significant (*p* < .05).

^a^ Data were collected via the Neuropace patient data management system.

## 4 DISCUSSION

sEEG is widely regarded as a mainstay tool for defining seizure‐onset zones in patients with focal DRE. sEEG is traditionally aimed at guiding resective or destructive procedures and is not routinely used to guide neuromodulation. Prior work has suggested that sEEG may improve outcomes when used to guide neuromodulation targeting. For example, Owens et al.^7^ reported markedly higher responder rates among patients who underwent sEEG before RNS implantation than in those who did not (82.3% vs 53.8%), positing that enhanced seizure‐onset zone localization may translate to better long‐term seizure control. Similarly, other studies have emphasized the importance of precise electrode targeting—often informed by sEEG—in predicting RNS benefit.^8^ Although this hypothesis is plausible, real‐world data have yet to demonstrate an improvement in seizure reduction with prior intracranial monitoring. In addition, RNS efficacy has been demonstrated to improve over time and overall seizure reduction likely relates to follow‐up duration.^10^ Our median follow‐up duration (3.8 ± .2 years) is slightly longer than the previously discussed studies (~2–3 years of follow‐up) but overall agrees with the trends observed in these studies, with greater seizure reduction over time. We also found that time to implantation was significantly longer for sEEG patients (6.9 vs 2.8 months), highlighting a potentially important barrier to care when invasive monitoring is pursued. However, this was not associated with seizure reduction outcomes (*p* = .756).

Our findings do not support a clinically meaningful difference in RNS outcomes between patients who did and did not undergo preoperative sEEG. In our cohort of 99 patients, median seizure reduction at last follow‐up was similar across groups (72% in the sEEG group vs 74% in the no‐sEEG group, *p* = .777). After propensity score matching, seizure reduction remained nearly identical (75% vs 74%, *p* = .914). These findings suggest that high‐quality video‐EEG monitoring alone may be sufficient for RNS targeting in appropriately selected patients. Because our no‐sEEG group had a high proportion of patients with bilateral TLE, this subgroup carries the strongest evidence for upfront RNS. However, we did not observe differences in seizure outcomes across the other epilepsy subtypes, suggesting that unilateral TLE arising from the dominant temporal lobe, temporal plus, and extratemporal epilepsy may be amenable to upfront RNS as well. This finding aligns with published series that emphasize long‐term progressive improvement in RNS therapy regardless of evaluation strategy.^6^, ^10^, ^11^ Furthermore, Sadeghzadeh et al.^12^ recently examined the Euclidean distance between the seizure‐onset zone (determined via sEEG) and the implanted RNS lead in neocortical epilepsy without any demonstrable relationship with seizure outcome at last follow‐up. Patients in their cohort achieved a median seizure reduction of 77.5%, which mirrors our findings. Although sEEG can provide crucial information in identifying the seizure‐onset zone and ultimately guiding RNS lead targeting, this may only partially identify the broader epileptogenic focus in some cases. In our cohort, at least one seizure‐onset zone was confidently identified in 95% of patients who underwent sEEG investigation. The distinction between the seizure‐onset zone and the broader epileptogenic focus may be a contributing factor to the lack of the relationship between sEEG utilization and overall seizure reduction in patients undergoing RNS implantation.

We observed some differences in epilepsy subtype between groups: unilateral TLE was more common in the sEEG cohort, whereas bilateral TLE was more common in the no‐sEEG cohort. Despite this, outcomes remained equivalent, consistent with prior literature showing comparable RNS efficacy across mesial temporal and neocortical foci.^7^, ^9^ Our findings therefore challenge the assumption that invasive localization is necessary for optimal outcomes in TLE. However, our findings may be subject to selection bias given the heterogeneity between these two patient populations. Unilateral non‐dominant TLE, in the pursuit of resective or ablative procedures, often prompts intracranial monitoring to rule out TLE mimickers and bilateral pathology and to establish any neocortical component. Meanwhile, bilateral TLE on scalp EEG, given the limited resective or ablative options, may dissuade multidisciplinary teams from intracranial monitoring. This possibility of selection bias is further compounded by the limited number of patients in each epilepsy subtype in the no‐sEEG cohort. This significantly limits our ability to detect true differences among groups and leaves our analyses subject to inherent heterogeneity among these patients.

Our multivariable regression highlights several factors associated with improved seizure reduction that extend beyond sEEG use. Greater age, female sex, thalamic RNS electrode implantation, and longer follow‐up were all associated with greater seizure reduction. The finding that thalamic stimulation was associated with improved outcomes aligns with emerging evidence supporting RNS targeting of thalamocortical networks.^13^, ^14^, ^15^ This may reflect broader network modulation rather than traditional seizure‐onset zone targeting, consistent with ongoing shifts toward network‐guided neuromodulation.

The mechanism of RNS efficacy is multifactorial, involving both acute seizure termination and chronic modulation of epileptogenic networks. Although initial hypotheses focused on direct inhibition of ictal activity, recent evidence suggests that long‐term network reorganization and plasticity are central to therapeutic benefit^16^: Patients with greater interictal network reorganization experience more substantial seizure reduction. Furthermore, the chronic recording capability of RNS provides ambulatory, naturalistic electrocorticography, which is virtually impossible to recreate with sEEG given the need for an associated inpatient hospital stay. Insights from these naturalistic recordings allow for a more nuanced, personalized approach to each patient's epilepsy subtype.^17^

### 4.1 Limitations

Our study has several limitations. The retrospective nature introduces potential selection bias, particularly in determining which patients underwent sEEG evaluation. Although propensity score matching mitigates some baseline differences, unmeasured confounders may remain. In addition, epilepsy subtype and anatomic heterogeneity may have influenced targeting strategies and outcomes and may not be fully captured by our categorical variables. Our sample of no‐sEEG patients was also comparatively small (*n* = 21), thereby limiting the statistical power to detect subtle differences. Although we reported the documented reasons these patients did not undergo intracranial monitoring, there may be nuanced discussions surrounding factors that we failed to capture in our analysis. Furthermore, although propensity score matching showed balanced groups, matching was performed using the measured variables. Therefore, unmeasured confounders remain unbalanced. This underscores the need for randomized data regarding the utility of intracranial monitoring in select patients. Finally, center‐specific expertise in scalp EEG interpretation and RNS programming may limit applicability to centers with different workflows or resource constraints.

## 5 CONCLUSION

In this large, single‐center cohort with long‐term follow‐up, preoperative sEEG evaluation did not significantly reduce seizure number or severity among patients undergoing RNS implantation. Seizure outcomes and patient‐reported improvements were comparable between patients whose targeting was guided by sEEG vs high‐quality vEEG alone, even after propensity matching. Factors associated with improved outcomes—older age, female sex, thalamic electrode placement, and longer follow‐up—highlight the importance of network‐level modulation and longitudinal optimization rather than seizure‐onset zone localization modality alone. These findings challenge the emerging assumption that invasive monitoring is beneficial for optimizing RNS efficacy in all patients and suggest that scalp EEG–guided targeting may be sufficient in appropriately selected cases. Future prospective studies are needed to clarify the role of sEEG in neuromodulation planning.

## AUTHOR CONTRIBUTIONS

Ben Shofty, David Botros, Julian Brown: Conception and design. Julian Brown and David Botros: Acquisition of data. David Botros and Ben Shofty: Analysis and interpretation of data. David Botros, Julian Brown, and Ben Shofty: Drafting the article. All authors: Reviewing and editing of article.

## FUNDING INFORMATION

The authors have nothing to report.

## CONFLICT OF INTEREST STATEMENT

None of the authors has any conflicts of interest to disclose.

## ETHICS STATEMENT

This study was approved by the University of Utah Institutional Review Board with a waiver of informed consent (#00115230). We confirm that we have read the Journal's position on issues involved in ethical publication and affirm that this report is consistent with those guidelines.

## Supporting information


**Figure S1:** Love plot demonstrating adjusted vs unadjusted absolute standardized mean differences of each variable included in the propensity score matching algorithm.

**Table S1:** Patient‐specific responsive neurostimulation constructs and seizure outcome data.

## Data Availability

The data that support the findings of this study are available from the corresponding author upon reasonable request.
