# Supplementary material for: Stereo‐electroencephalography–guided responsive neuromodulation: A propensity‐matched cohort study
Source: Epilepsia. 2026 Jul 14;67(10):5179–88. doi: 10.1002/epi.70377 (PMC13675770; doi:10.1002/epi.70377)
Supplement: Supplementary file 1 [file EPI-67-5179-s001.docx]

**Supplemental Table 1. Patient-specific RNS Constructs and Seizure Outcome Data**

| ID | Age (y) | Sex | Epilepsy duration (y) | Seizure types | Preop seizures/ month | # of prior intervention(s) | VNS | Preop SEEG | Epilepsy subtype | Lesion | Lesion type | Lesion location | Preop # of ASMs | RNS target | | Additional targets or procedures | Seizure reduction (% at last follow up) | Current # of ASMs | PDMS seizure reduction (%) | Severity |
| --- | --- | --- | --- | --- | --- | --- | --- | --- | --- | --- | --- | --- | --- | --- | --- | --- | --- | --- | --- | --- |
|  |  |  |  |  |  |  |  |  |  |  |  |  |  | Target 1 | Target 2 |  |  |  |  |  |
| 1 | 38.35 | F | 34 | FIAS | 30 | 2 | No | Yes | Temporal plus | Yes | Gliosis | Right frontal operculum, anterior left frontal lobe | 3 | R SFG (SE) | R STG (SE) | Partial right frontal lobectomy | 100 | 3 | 100 |  |
| 2 | 40.73 | F | 3 | FIAS, SGTC | 3 | 0 | No | Yes | Unilateral TLE | No |  |  | 3 | L HIP (D) | R HIP (D) |  | 67 | 3 | 90-99 | Better |
| 3 | 21.74 | F | 16 | FIAS, SGTC | 2 | 1 | No | Yes | Temporal plus | Yes | Gliosis | Right temporal | 2 | L CM (D) | R CM (D) |  | 0 | 2 | 90-99 | Better |
| 4 | 42.34 | F | 18 | FIAS, SGTC | 4 | 0 | No | Yes | Unilateral TLE | No |  |  | 2 | L HIP (D) | R HIP (D) |  | 88 | 2 | 90-99 | Better |
| 5 | 50.15 | M | 2 | FIAS, SGTC | 2 | 0 | No | Yes | Bilateral TLE | No |  |  | 1 | L HIP (D) | R HIP (D) |  | 100 | 1 | 1-24 | Better |
| 6 | 42.46 | F | 4 | FIAS, SGTC | 12 | 0 | No | Yes | Unilateral TLE | No |  |  | 2 | L HIP (D) | L subtemporal (SE) |  | 100 | 2 | 90-99 |  |
| 7 | 66.99 | F | 36 | FIAS, SGTC | 4 | 0 | No | Yes | Temporal plus | No |  |  | 3 | L HIP (D) | R HIP (D) |  | 75 | 3 | 50-74 | Better |
| 8 | 23.88 | F | 9 | FIAS, SGTC | 90 | 1 | Yes | No | Extratemporal | Yes | MTS | Right anterior temporal | 4 | L HIP (D) | R HIP (D) |  | 87 | 4 | 75-89 | Much better |
| 9 | 2.53 | F | 27 | FIAS, SGTC | 30 | 1 | No | Yes | Temporal plus | No |  |  | 2 | L HIP (D) | R HIP (D) |  | 47 | 2 | 50-74 | Better |
| 10 | 21.48 | M | 15 | FIAS, SGTC | 30 | 0 | No | Yes | Extratemporal | No |  |  | 3 | L temporal (SE) | L occipital (SE) | Resection of left parietal focus | 33 | 4 | 25-49 | Better |
| 11 | 31.74 | F | 16 | FIAS, SGTC | 12 | 1 | No | No | Bilateral TLE | Yes | Gliosis | Left temporal | 3 | L HIP (D) | R HIP (D) |  | 67 | 3 | 50-74 | Better |
| 12 | 20.40 | F | 8 | FIAS, SGTC | 4 | 1 | No | Yes | Extratemporal | Yes | FCD | Left temporal | 3 | R SFG (SE) | L frontal interhemispheric (SE) |  | 50% | 3 | 25-49 | Better |
| 13 | 38.44 | M | 30 | FIAS, SGTC | 20 | 2 | No | No | Extratemporal | No |  |  | 3 | L CM (D) | R CM (D) |  | 0 | 3 | 25-49 | Better |
| 14 | 20.10 | M | 7 | FIAS, SGTC | 30 | 0 | No | No | Temporal plus | Yes | FCD | Left anterior temporal | 2 | L CM (D) | R CM (D) |  | 97 | 2 | 25-49 | Better |
| 15 | 36.34 | M | 35 | FIAS, SGTC | 60 | 0 | No | Yes | Unilateral TLE | No |  |  | 2 | L ANT (D) | R ANT (D) |  | 100 | 3 | 75-89 | Better |
| 16 | 40.64 | M | 2 | FIAS, SGTC | 5 | 1 | No | Yes | Temporal plus | No |  |  | 2 | L HIP (D) | R HIP (D) |  | 80 | 2 | 25-49 | Better |
| 17 | 23.96 | M | 2 | FIAS, SGTC | 30 | 0 | No | Yes | Extratemporal | No |  |  | 2 | R SFG (SE) | R MFG (SE) | R IFG (SE) | 0 | 2 | 75-89 |  |
| 18 | 25.31 | M | 24 | FIAS, SGTC | 10 | 1 | No | No | Temporal plus | No |  |  | 2 | R temporal (SE) | L parietal (SE) |  | 80 | 2 | 75-89 | Much better |
| 19 | 23.41 | F | 16 | FIAS, SGTC | 150 | 1 | Yes | Yes | Extratemporal | No |  |  | 3 | R HIP (D) | R precentral (SE) |  | 80 | 3 | 90-99 |  |
| 20 | 26.09 | F | 8 | FIAS, SGTC | 30 | 1 | Yes | Yes | Unilateral TLE | No |  |  | 2 | L subfrontal (SE) | L temporal (SE) |  | 97 | 3 | 50-74 | Better |
| 21 | 35.77 | M | 3 | FIAS, SGTC | 60 | 0 | No | Yes | Bilateral TLE | No |  |  | 1 | L HIP (D) | R HIP (D) |  | 100 | 1 | 75-89 | Better |
| 22 | 24.39 | M | 15 | FIAS, SGTC | 2 | 0 | No | Yes | Unilateral TLE | No |  |  | 3 | L HIP (D) | R HIP (D) |  | 50 | 3 | 1-24 | Better |
| 23 | 23.89 | M | 14 | FIAS, SGTC | 4 | 0 | No | Yes | Unilateral TLE | No |  |  | 2 | L HIP (D) | R HIP (D) |  | 50 | 2 | 25-49 | Better |
| 24 | 42.11 | F | 12 | FIAS | 3 | 0 | No | No | Bilateral TLE | No |  |  | 2 | L HIP (D) | R HIP (D) |  | 100 | 2 | 50-74 | Better |
| 25 | 43.78 | F | 39 | FIAS | 25 | 1 | Yes | No | Temporal plus | Yes | Heterotopia | Left hemispheric | 3 | L CM (D) | R CM (D) |  | 100 | 3 | 100 | No Change |
| 26 | 24.93 | M | 10 | FIAS, SGTC | 3 | 0 | No | Yes | Unilateral TLE | No |  |  | 3 | L frontal (SE) | L STG (SE) |  | 33 | 2 | 25-49 | Better |
| 27 | 29.38 | F | 8 | FIAS | 3 | 0 | No | Yes | Extratemporal | No |  |  | 2 | L precentral (SE) | L ITG (SE) |  | 33 | 2 | 0 |  |
| 28 | 46.35 | M | 44 | FIAS | 30 | 2 | Yes | Yes | Bilateral TLE | No |  |  | 2 | L HIP (D) | R temporal (SE) | Right temporal lobectomy | 100 | 3 | 100 | No Change |
| 29 | 45.35 | F | 34 | FAS, SGTC | 25 | 0 | No | Yes | Temporal plus | Yes | Gliosis | Right frontal, temporal, and L anterior temporal | 2 | L HIP (D) | R HIP (D) |  | 100 | 3 | 90-99 |  |
| 30 | 49.79 | M | 16 | FIAS, SGTC | 30 | 0 | No | Yes | Bilateral TLE | No |  |  | 2 | L HIP (D) | R HIP (D) |  | 91 | 2 | 0 | No Change |
| 31 | 31.60 | F | 16 | FIAS, SGTC | 30 | 0 | No | No | Temporal plus | Yes | MTS | Left mesial temporal sclerosis | 3 | L HIP (D) | L subtemporal (SE) |  | 60 | 3 | 50-74 | Better |
| 32 | 30.98 | F | 13 | FAS, SGTC | 150 | 0 | No | Yes | Unilateral TLE | No |  |  | 3 | L parietal (SE) | L parietal (SE) |  | 99 | 2 | 50-74 | Better |
| 33 | 29.68 | M | 8 | FIAS, SGTC | 30 | 0 | No | Yes | Unilateral TLE | Yes | Tumor | Left temporal multinodular evacuating neuronal tumor | 1 | L HIP (D) | L STG (SE) | Left temporal biopsy | 83 | 1 | 90-99 | Better |
| 34 | 20.88 | F | 15 | GTC | 10 | 1 | Yes | No | Extratemporal | No |  |  | 2 | L CM (D) | R CM (D) |  | 100 | 2 | 100 | Much better |
| 35 | 21.57 | M | 2 | FIAS, SGTC | 6 | 0 | No | Yes | Temporal plus | No |  |  | 1 | L HIP (D) | R HIP (D) |  | 100 | 1 | 75-89 |  |
| 36 | 24.98 | F | 16 | FAS, SGTC | 1 | 0 | No | Yes | Temporal plus | No |  |  | 1 | L HIP (D) | R HIP (D) |  | 100 | 1 | 90-99 | Much better |
| 37 | 38.36 | M | 17 | FIAS, SGTC | 12 | 1 | No | Yes | Unilateral TLE | No |  |  | 3 | L HIP (D) | L ANT (D) |  | 75 | 3 | 50-74 | Better |
| 38 | 26.57 | F | 23 | FIAS, SGTC | 30 | 1 | Yes | Yes | Extratemporal | Yes | Gliosis | Left MCA distribution | 4 | L temporal (SE) | L temporal (SE) | Resection of left sensory motor focus | 73 | 4 | 50-74 | Better |
| 39 | 50.60 | M | 37 | FIAS, SGTC | 2 | 0 | No | Yes | Unilateral TLE | Yes | MTS | Left MTS | 1 | L HIP (D) | R HIP (D) |  | 50 | 1 | 50-74 | Better |
| 40 | 70.16 | F | 3 | FIAS | 60 | 0 | No | No | Bilateral TLE | Yes | MTS | Left MTS | 2 | L HIP (D) | R HIP (D) |  | 80 | 1 | 75-89 | Better |
| 41 | 57.90 | F | 55 | FIAS, SGTC | 4 | 0 | No | No | Bilateral TLE | No |  |  | 2 | L HIP (D) | R HIP (D) |  | 75 | 2 | 75-89 | Better |
| 42 | 40.04 | F | 13 | FAS, SGTC | 5 | 1 | Yes | Yes | Unilateral TLE | No |  |  | 4 | L HIP (D) | L temporal (SE) |  | 100 | 2 | 100 |  |
| 43 | 43.48 | M | 16 | FIAS, SGTC | 4 | 1 | Yes | Yes | Unilateral TLE | No |  |  | 2 | L HIP (D) | R HIP (D) | VNS battery replacement | 100 | 2 | 50-74 | Better |
| 44 | 33.88 | M | 17 | FAS, SGTC | 2 | 0 | No | Yes | Extratemporal | No |  |  | 2 | R CM (D) | R IFG (SE) |  | 79 | 2 | 75-89 | Better |
| 45 | 18.99 | F | 9 | FAS, SGTC | 90 | 0 | No | Yes | Extratemporal | No |  |  | 1 | L parietal (SE) | L parietal (SE) |  | 89 | 1 | 75-89 | Better |
| 46 | 30.98 | F | 17 | FAS | 150 | 0 | No | Yes | Extratemporal | No |  |  | 3 | R frontal interhemispheric (SE) | R frontal interhemispheric (SE) |  | 96 | 3 | 90-99 |  |
| 47 | 20.28 | M | 19 | FIAS | 20 | 1 | Yes | Yes | Unilateral TLE | Yes | MTS | Bilateral mesial temporal | 4 | L HIP (D) | R HIP (D) |  | 25 | 4 | 75-89 | Better |
| 48 | 39.14 | F | 7 | FIAS, SGTC | 4 | 0 | No | No | Bilateral TLE | No |  |  | 3 | L HIP (D) | R HIP (D) |  | 75 | 4 | 50-74 |  |
| 49 | 31.23 | F | 15 | FIAS, SGTC | 4 | 0 | No | Yes | Temporal plus | No |  |  | 2 | R CM (D) | R HIP (D) |  | 100 | 3 | 100 | Better |
| 50 | 35.46 | M | 27 | FIAS, SGTC | 30 | 2 | Yes | Yes | Extratemporal | No |  |  | 3 | R precentral (SE) | R parietal (SE) |  | 0 | 3 | 50-74 | Better |
| 51 | 24.94 | M | 7 | FIAS, SGTC | 4 | 0 | No | Yes | Bilateral TLE | Yes | FCD | Left temporal | 2 | L HIP (D) | R HIP (D) |  | 100 | 2 | 75-89 | Much better |
| 52 | 24.86 | M | 2 | FIAS, SGTC | 2 | 0 | No | No | Unilateral TLE | Yes | Gliosis | Right frontotemporal | 2 | L HIP (D) | R HIP (D) |  | 50 | 2 | 25-49 | Better |
| 53 | 30.18 | F | 4 | FAS, SGTC | 6 | 0 | No | Yes | Unilateral TLE | No |  |  | 2 | L HIP (D) | R HIP (D) |  | 100 | 1 | 100 | Better |
| 54 | 30.77 | F | 18 | FIAS | 5 | 1 | Yes | Yes | Unilateral TLE | No |  |  | 3 | L HIP (D) | R HIP (D) |  | 100 | 3 | 50-74 | Better |
| 55 | 53.66 | M | 9 | FIAS, SGTC | 30 | 1 | Yes | Yes | Bilateral TLE | No |  |  | 3 | L HIP (D) | R HIP (D) |  | 80 | 3 | 50-74 | Better |
| 56 | 61.59 | F | 40 | FAS, SGTC | 30 | 0 | No | No | Bilateral TLE | No |  |  | 3 | L HIP (D) | L subtemporal (SE) |  | 50 | 3 | 25-49 | Better |
| 57 | 47.59 | M | 2 | FIAS, SGTC | 2 | 0 | No | Yes | Unilateral TLE | No |  |  | 2 | L HIP (D) | R HIP (D) |  | 50 | 3 | 50-74 | Better |
| 58 | 40.26 | F | 27 | FIAS, SGTC | 3 | 0 | No | Yes | Bilateral TLE | No |  |  | 2 | L HIP (D) | R HIP (D) |  | 100 | 3 | 50-74 | Better |
| 59 | 49.52 | M | 31 | FIAS, SGTC | 10 | 0 | No | No | Bilateral TLE | No |  |  | 2 | L HIP (D) | R HIP (D) |  | 80 | 2 | 50-74 | Better |
| 60 | 34.85 | M | 18 | FIAS, SGTC | 4 | 0 | No | Yes | Unilateral TLE | No |  |  | 3 | L HIP (D) | R HIP (D) |  | 50 | 3 | 25-49 | Better |
| 61 | 50.75 | M | 50 | FIAS, SGTC | 30 | 1 | Yes | Yes | Unilateral TLE | No |  |  | 4 | L HIP (D) | R HIP (D) |  | 20 | 4 | 1-24 | Better |
| 62 | 21.37 | F | 2 | FIAS, SGTC | 4 | 0 | No | Yes | Temporal plus | No |  |  | 3 | L MFG (SE) | L posterior frontal (SE) |  | 100 | 2 | 75-89 | Better |
| 63 | 29.68 | M | 16 | FIAS, SGTC | 24 | 1 | Yes | Yes | Bilateral TLE | Yes | Heterotopia | Bilateral periventricular | 3 | L anterior temporal (SE) | L posterior temporal (SE) |  | 50 | 3 | 50-74 | Better |
| 64 | 42.99 | F | 22 | FIAS, SGTC | 6 | 0 | No | No | Unilateral neocortical TLE | Yes | Gliosis | Right parietal | 3 | L HIP (D) | L STG (SE) |  | 100 | 3 | 90-99 |  |
| 65 | 36.45 | M | 27 | FIAS, SGTC | 30 | 0 | No | Yes | Temporal plus | No |  |  | 2 | L ANT (D) | L CM (D) |  | 100 | 2 | 25-49 | Better |
| 66 | 21.74 | F | 14 | FIAS, SGTC | 30 | 0 | No | Yes | Temporal plus | No |  |  | 3 | L ANT (D) | R ANT (D) |  | 97 | 3 | 75-89 | Better |
| 67 | 50.79 | M | 33 | FAS, SGTC | 5 | 2 | Yes | Yes | Temporal plus | No |  |  | 3 | L VENTRAL PFC (SE) | L CM (D) |  | 100 | 2 | 90-99 | Better |
| 68 | 26.39 | F | 10 | FIAS, SGTC | 60 | 1 | Yes | Yes | Bilateral TLE | No |  |  | 4 | L HIP (D) | R HIP (D) |  | 50 | 5 | 50-74 | Better |
| 69 | 37.49 | M | 21 | FAS, SGTC | 2 | 2 | Yes | Yes | Bilateral TLE | No |  |  | 2 | L HIP (D) | R HIP (D) |  | 100 | 4 | 100 |  |
| 70 | 25.14 | M | 25 | FIAS | 3 | 0 | No | Yes | Unilateral TLE | Yes | Gliosis | Left frontoparietal gliosis | 2 | L CM (D) | L IFG (SE) |  | 100 | 2 | 75-89 | Better |
| 71 | 40.51 | M | 27 | FIAS, SGTC | 10 | 2 | Yes | Yes | Unilateral TLE | No |  |  | 3 | L HIP (D) | R posterior temporal (SE) |  | 40 | 3 | 50-74 | Better |
| 72 | 19.79 | F | 5 | FIAS, SGTC | 3 | 0 | No | Yes | Unilateral TLE | No |  |  | 2 | L HIP (D) | R HIP (D) |  | 33 | 2 | 25-49 | Better |
| 73 | 24.45 | M | 4 | FAS, SGTC | 12 | 0 | No | Yes | Bilateral TLE | No |  |  | 2 | L HIP (D) | R HIP (D) |  | 42 | 2 | 25-49 | Better |
| 74 | 66.70 | F | 5 | FIAS, SGTC | 8 | 0 | No | Yes | Temporal plus | No |  |  | 2 | L HIP (D) | R HIP (D) |  | 88 | 1 | 25-49 | Better |
| 75 | 46.53 | F | 20 | FIAS, SGTC | 20 | 1 | Yes | Yes | Unilateral TLE | No |  |  | 5 | L CM (D) | R HIP (D) |  | 80 | 4 | 90-99 | Much better |
| 76 | 22.26 | M | 15 | FIAS, SGTC | 16 | 0 | No | Yes | Temporal plus | No |  |  | 1 | L HIP (D) | R CM (D) |  | 25 | 1 | 25-49 | Better |
| 77 | 38.37 | F | 30 | FIAS, SGTC | 12 | 1 | No | Yes | Bilateral TLE | Yes | FCD | Right frontal | 3 | L HIP (D) | R HIP (D) |  | 92 | 3 | 50-74 | Better |
| 78 | 31.70 | M | 10 | FIAS, SGTC | 2 | 0 | No | Yes | Bilateral TLE | No |  |  | 3 | L HIP (D) | R HIP (D) |  | 50 | 3 | 50-74 | Better |
| 79 | 58.15 | M | 15 | FIAS, SGTC | 4 | 1 | No | Yes | Unilateral TLE | No |  |  | 2 | L parietal (SE) | L anterior temporal (SE) |  | 75 | 2 | 50-74 | Better |
| 80 | 50.70 | M | 30 | FIAS | 16 | 1 | Yes | Yes | Unilateral TLE | Yes | Heterotopia | Right temporal | 4 | R CM (D) | R posterior temporal (SE) |  | 81 | 3 | 75-89 | Much better |
| 81 | 25.09 | M | 2 | FAS, SGTC | 4 | 1 | No | Yes | Extratemporal | No |  |  | 4 | R parietooccipital (SE) | R frontoparietal (SE) | Right parietal seizure focus resection | 75 | 4 | 90-99 |  |
| 82 | 27.60 | F | 9 | FIAS, SGTC | 12 | 0 | No | Yes | Temporal plus | No |  |  | 2 | R HIP (D) | R anterior temporal (SE) |  | 92 | 2 | 100 |  |
| 83 | 34.67 | M | 15 | FIAS, SGTC | 20 | 0 | No | Yes | Unilateral TLE | Yes | Gliosis | Left temporal | 2 | L MTG (SE) | L posterior temporal (SE) |  | 0 | 1 | 25-49 | Better |
| 84 | 59.35 | M | 40 | FIAS, SGTC | 12 | 0 | No | No | Unilateral TLE | No |  |  | 3 | L HIP (D) | L STG (SE) |  | 67 | 2 | 100 |  |
| 85 | 26.90 | M | 12 | FIAS, SGTC | 15 | 0 | No | Yes | Extratemporal | No |  |  | 3 | L HIP (D) | L frontoparietal (SE) |  | 73 | 3 | 50-74 | Better |
| 86 | 52.89 | F | 40 | FIAS, SGTC | 30 | 0 | No | Yes | Extratemporal | No |  |  | 4 | L CM (D) | R CM (D) |  | 93 | 3 | 90-99 | Better |
| 87 | 30.31 | F | 19 | FIAS, SGTC | 30 | 1 | Yes | Yes | Extratemporal | Yes | Gliosis | Right frontal | 2 | R parietal (SE) | R temporal (SE) | Resection of right premotor/ cingulate seizure focus | 97 | 2 | 90-99 | Better |
| 88 | 43.05 | F | 40 | FIAS, SGTC | 60 | 0 | No | Yes | Bilateral TLE | No |  |  | 2 | L HIP (D) | R HIP (D) |  | 50 | 2 | 50-74 | Better |
| 89 | 33.14 | M | 32 | FAS | 30 | 2 | Yes | Yes | Temporal plus | No |  |  | 3 | R paracentral lobule (SE) | R precentral (SE) |  | 50 | 3 | 50-74 | Better |
| 90 | 38.15 | M | 14 | FIAS, SGTC | 15 | 0 | No | No | Bilateral TLE | Yes | MTS | Right mesial temporal | 2 | L HIP (D) | R HIP (D) |  | 73 | 4 | 90-99 | Better |
| 91 | 42.04 | F | 14 | FIAS, SGTC | 30 | 0 | No | Yes | Extratemporal | No |  |  | 3 | R parietal (SE) | R temporal (SE) |  | 100 | 3 | 75-89 | Better |
| 92 | 33.97 | M | 5 | FIAS, SGTC | 4 | 1 | No | Yes | Bilateral TLE | No |  |  | 2 | L HIP (D) | R HIP (D) |  | 75 | 2 | 90-99 |  |
| 93 | 17.98 | M | 2 | FIAS, SGTC | 8 | 0 | No | No | Unilateral TLE | No |  |  | 2 | L HIP (D) | L anterior temporal (SE) | L subtemporal (SE) | 13 | 2 | 90-99 | Better |
| 94 | 29.62 | M | 21 | FIAS, SGTC | 16 | 1 | Yes | No | Unilateral neocortical TLE | No |  |  | 2 | L HIP (D) | R HIP (D) |  | 94 | 3 | 90-99 | Better |
| 95 | 49.98 | M | 26 | FIAS, SGTC | 2 | 0 | No | No | Extratemporal | No |  |  | 3 | L CM (D) | R CM (D) |  | 100 | 2 | 25-49 | No change |
| 96 | 21.72 | M | 17 | FIAS, SGTC | 30 | 2 | Yes | Yes | Bilateral TLE | Yes | Gliosis | Left MCA distribution | 4 | L HIP (D) | R HIP (D) |  | 80 | 4 | 75-89 | Better |
| 97 | 25.06 | M | 5 | FIAS, SGTC | 12 | 0 | No | Yes | Unilateral TLE | No |  |  | 3 | L HIP (D) | L temporal (SE) |  | 92 | 3 | 90-99 | Much better |
| 98 | 23.05 | M | 10 | FIAS | 12 | 0 | No | Yes | Temporal plus | No |  |  | 3 | L HIP (D) | R HIP (D) |  | 67 | 3 | 25-49 | Better |
| 99 | 38.28 | M | 26 | FIAS, SGTC | 12 | 0 | No | Yes | Unilateral TLE | No |  |  | 3 | L HIP (D) | R HIP (D) |  | 0 | 3 | 50-74 | Better |

VNS, vagal nerve stimulation; SEEG, stereoelectroencephalography; ASM, antiseizure medication; PDMS, patient data management system; FIAS: focal impaired awareness seizure; SE: surface electrode; D: depth electrode; SGTC, secondary generalized tonic clonic; FAS focal aware seizure; GTC, generalized tonic clonic; TLE, temporal lobe epilepsy; MTS, mesial temporal sclerosis; FCD, focal cortical dysplasia; MCA, middle cerebral artery; SFG, superior frontal gyrus; STG, superior temporal gyrus; HIP, hippocampus; CM, centromedian nucleus of the thalamus; ANT, anterior nucleus of the thalamus; MFG, middle frontal gyrus; ITG, inferior temporal gyrus
